# Complexities of Long-Term Care With Gastro-Jejunal (GJ) Feeding Tubes and Enteral Migration During COVID-19 Pandemic Times: A Case Report

**DOI:** 10.7759/cureus.27870

**Published:** 2022-08-11

**Authors:** Mansoor Zafar, Florence Saddler, Joe Parvin, Eleanor Hennebry, Rayanna Pereira, Mark Austin

**Affiliations:** 1 Gastroenterology, Hepatobiliary, Hepatology, Royal Sussex County Hospital, University Hospitals Sussex NHS Foundation Trust, Brighton, GBR; 2 Infectious Diseases and General Internal Medicine, Royal Sussex County Hospital, University Hospital Sussex NHS Foundation Trust, Brighton, GBR; 3 General Internal Medicine and Gastroenterology, Royal Sussex County Hospital, University Hospitals Sussex NHS Foundation Trust, Brighton, GBR; 4 General Internal Medicine and Gastroenterology, Royal Sussex County Hospital, University Hospital Sussex NHS Foundation Trust, Brighton, GBR; 5 General Internal Medicine, Royal Sussex County Hospital, University Hospital Sussex NHS Foundation Trust, Brighton, GBR; 6 Gastroenterology and Hepatology and General Internal Medicine, Royal Sussex County Hospital, University Hospital Sussex NHS Foundation Trust, Brighton, GBR

**Keywords:** gastroparesis, delayed gastric emptying, autistic spectrum disorder, ehlers-danlos syndrome, covid-19, gastro-jejunal feeding tubes

## Abstract

Gastro-jejunostomy tubes, or percutaneous endoscopic gastrostomy tubes with jejunal extension (PEG-J), hold a significant role in the long-term nutritional management of patients with poor oral intake. This can be for a variety of reasons; ranging from metabolic conditions, including diabetes mellitus, inherited or congenital conditions like Ehler Danlos syndrome, or patients with neurological disorders, such as stroke, advanced Parkinson's disease or multiple sclerosis. Although they are very helpful for the overall nutritional needs of such patients, they are associated with complications, including the dislodging of jejunal tubes. The need to promptly recognise, investigate and manage this, in a timely manner, is vital, particularly during the COVID-19 pandemic times, as such patients may be associated with multiple comorbidities.

## Introduction

Gastro-jejunal (GJ) feeding tubes are inserted in patients with poor voluntary feeding. The common conditions associated with this include post-stroke [1], multiple sclerosis, and gastroparesis. Gastroparesis is usually a result of autonomic neuropathy, for example, long-standing diabetes mellitus, or inherited disorders, most commonly Ehler Danlos syndrome [2]. Gomes et al. carried out a systematic review and concluded that there is a significant benefit for a percutaneous feeding tube, as opposed to naso-gastric tubes, in the long-term care of patients with poor oral intake [3].

## Case presentation

An 18-year-old woman, wheelchair-bound and with a background history of Ehlers-Danlos syndrome associated with hypermobility; autistic spectrum disorder; and a significant history of gastro-oesophageal reflux disease was referred to the medical team from the emergency department (ED). Due to previous diagnoses of delayed gastric emptying and gastroparesis with chronic constipation, she previously had an endoscopically inserted percutaneous endoscopic gastrostomy (PEG) by the gastroenterology team. This had been supplemented with a jejunal extension of the PEG tube (a GJ) tube by the interventional radiology team, with a nutritional prescription for slow feeding over 17 hours. She had presented with a queried non-functioning GJ tube for five days in the community.

On arrival to ED, it was discovered that she had a positive lateral flow COVID-19 test five days previously in the community; however, screening on arrival with a polymerase chain reaction (PCR) COVID-19 test in ED was negative. Her observations (vital signs) were all within normal ranges, except for a weight of 55 kilograms (kg), and a body mass index (BMI) of 19. Her medications included lactulose 30 mL once a day (OD), solifenacin succinate 10 mg OD, cholecalciferol 800 units OD and gabapentin 300 mg OD.

The flushing of the feeding jejunal port resulted in the quick flow back from the gastric venting port. She also reported nausea and reflux symptoms. An impression of a bypassing or non-functional jejunal port was made. The patient was admitted to a side room in the COVID-19 ward and blood tests, including refeeding blood (electrolytes including adjusted calcium, phosphate and magnesium), were requested.

Due to COVID-19 pandemic safety concerns, the patient continued to be managed with intravenous fluid resuscitation, and daily refeeding blood and serum electrolyte assessments followed by replacements. During this time, multiple attempts were made to communicate with the radiology team to find a slot to have further imaging to assess.

**Figure 1:**
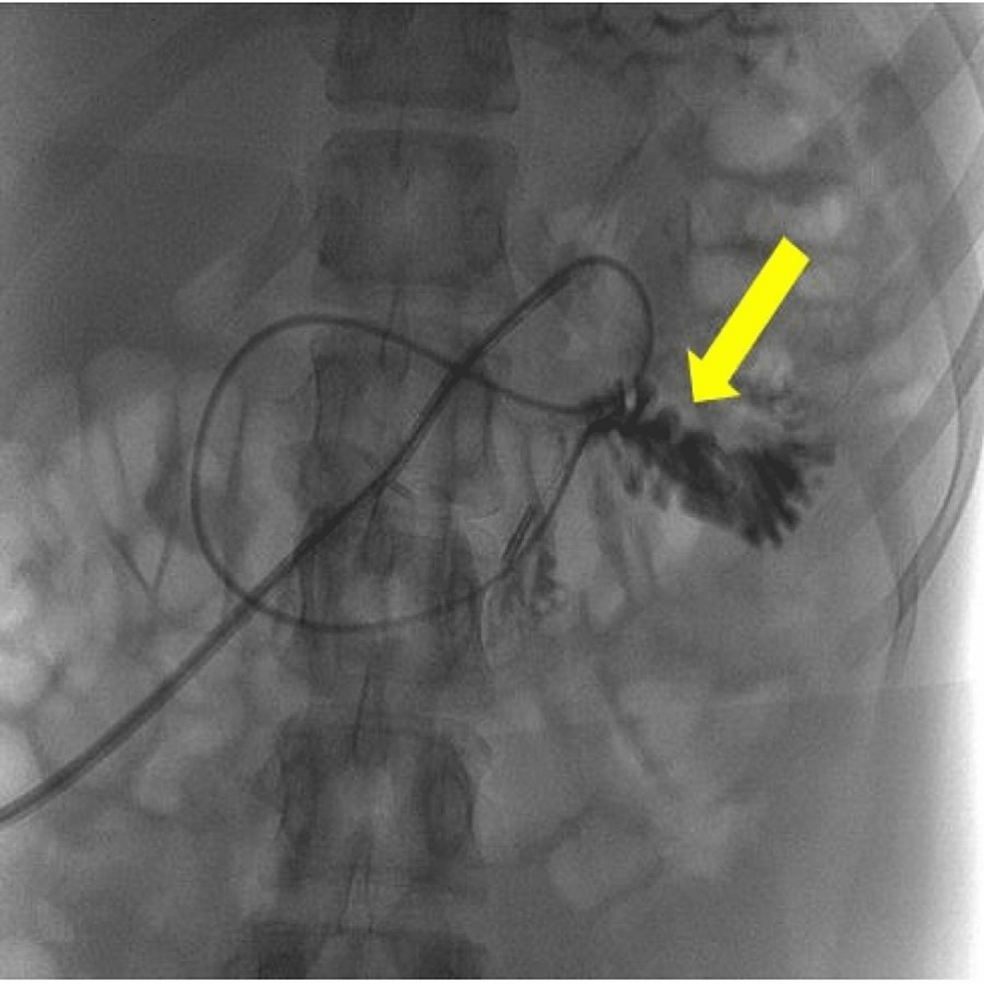
Contrast tubogram showing the tip of the jejunal extension has curled on itself at the duodeno-jejunal (DJ) flexure (yellow arrow). The inner (jejunal) tubing is not present within the external PEG tubing (external to the patient). Impression likely disconnected with contrast seen in both the stomach and the jejunum.

Eventually, two days later, a contrast tubogram showed that the jejunal extension had curled on itself at the duodeno-jejunal (DJ) flexure (Figure 1).

**Figure 2:**
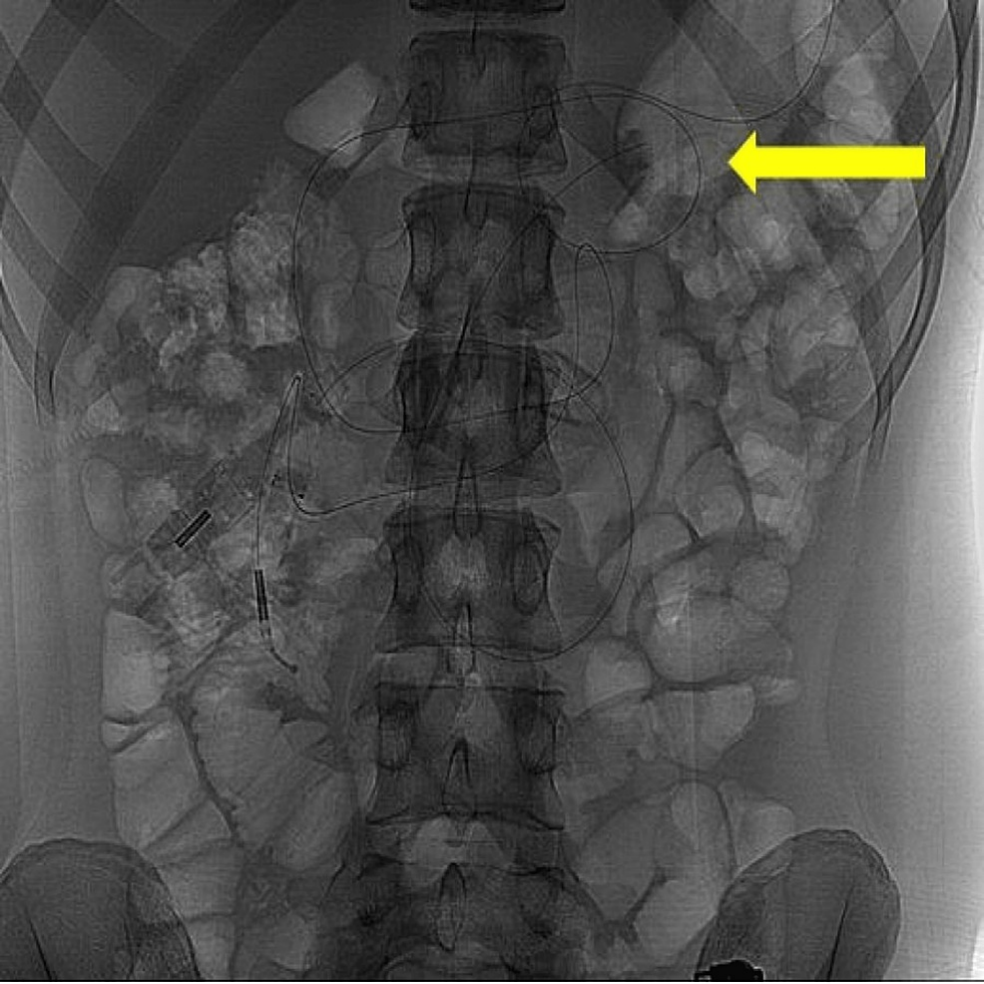
Fluoroscopic view of the abdomen-pelvis. The jejunal extension shows curled up segment within the stomach (yellow arrow).

Following this, a fluoroscopic view of the abdomen and pelvis was also requested, which confirmed the curled-up segment of the jejunal tube within the stomach (Figure 2).

While admitted to the side-room in the COVID-19 ward the patient was converted to a slower rate of feeding to avoid reflux symptoms, and endoscopy was booked five days later (in accordance with local hospital guidelines of endoscopy 10 days after positive lateral flow test for Covid-19) to locate the disconnected internal jejunal feeding tube. Additionally, the patient was started on slow intravenous fluids to maintain hydration. A decision was made to proceed to gastroscopy on day 11 (of positive lateral flow as per the local hospital guidelines) and attempt to retrieve the jejunal tube from the stomach.

**Figure 3:**
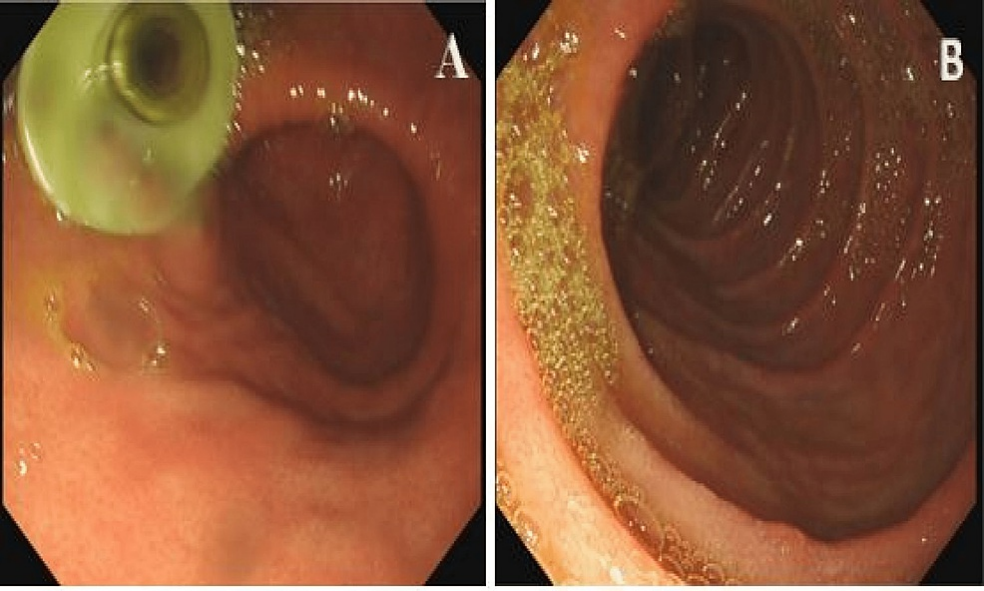
(A) Stomach in retroflexion. The PEG portion of the GJ tube is clearly visible within the stomach. (B) The jejunal tube is not present, despite visualising the jejunum (70cm past the pylorus). It could not be found and thus likely had passed distally.

 However, gastroscopy revealed no jejunal tube within the stomach, visualising to the jejunum (70cm past the pylorus). It could not be found and thus an impression of distal tube migration beyond the antrum was made (Figures 3A, 3B).

**Figure 4:**
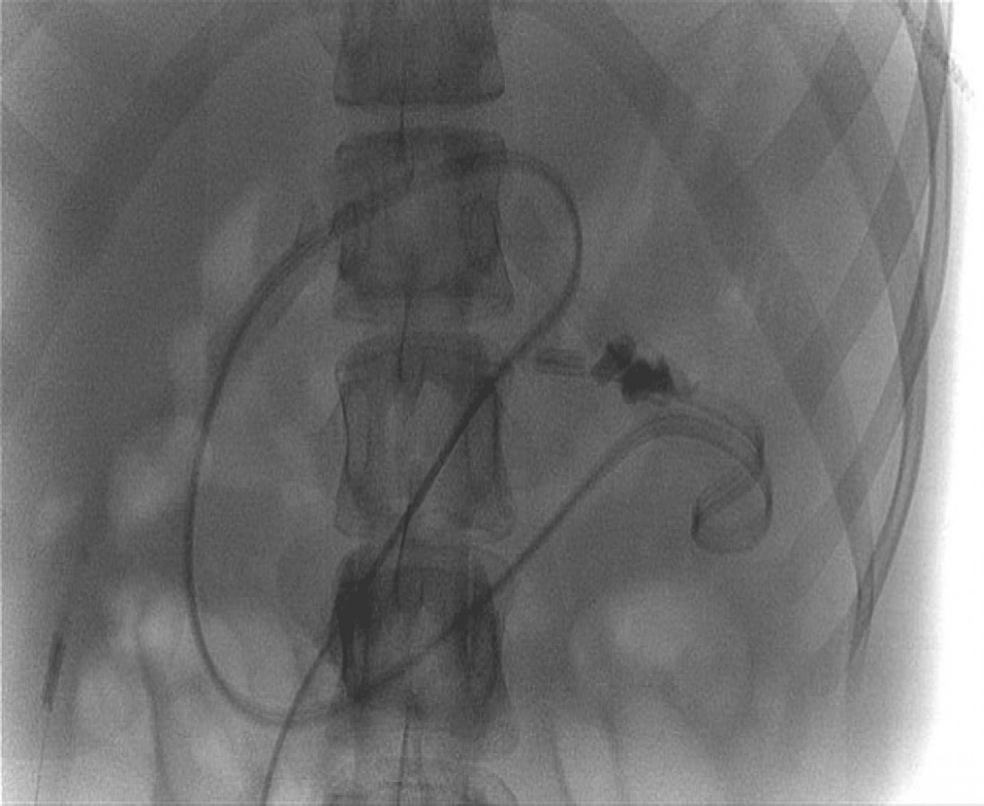
New freak-jejunal extension placed in a good position and securely attached to the gastrostomy

Following a multidisciplinary approach, the interventional radiology (IR) team successfully inserted a new jejunal (JEJ) extension to the PEG and advised it was safe to feed through (Figure 4).

A conservative approach considering the comorbidities was decided in a multi-disciplinary meeting for previously dislodged jejunal tube with distal enteral migration. The patient had serial abdominal x-ray with distal enteral migration of the jejunal tube, and remained asymptomatic throughout. The jejunal extension tube eventually passed out from the rectum on day 16 of positive lateral flow (day 11 of the admission to the hospital), which was confirmed with a repeated plain abdominal x-ray. The patient was eventually discharged back to the community.

## Discussion

For patients requiring alternative routes of nutrition estimated to be more than 30 days, it is a common practice to consider either PEG or GJ tubes [4]. Kurien et al. have demonstrated lower mortality rates in patients who have undergone gastrostomy procedures as opposed to those who have not [5].

Much has been written about the indications and management of gastrostomy tubes, including PEG or GJ tubes [6]. The complications include leakage [6], bleeding [7], and formation of fistulas including gastro-colo-cutaneous, colo-cutaneous or gastro-colic [8,9]. A buried bumper syndrome is another complication, which involves the migration of the internal bumper. This may be complete, with migration outside the gastrointestinal (GI) lumen to anywhere along the GI tract, or incomplete, where it can impact the gastric wall. Even though this syndrome can be diagnosed clinically, it would ideally need gastroscopy to confirm displacement [10,11]. Additional metabolic complications include refeeding syndrome. This is due to nutritional deficiencies associated with electrolyte loss and fluid shifts, predominantly deficiencies of phosphate, which is associated with cardiac and respiratory consequences [12,13]. The prompt monitoring of daily refeeding blood, with replacement of fluid and electrolytes, is of the utmost importance throughout the planning process for insertion of a PEG or GJ tube. This emphasises the need for prompt recognition, appropriate investigations, and efficient clinical management. In addition, understanding and confidence in replacing the removed gastrostomy tube, even with a Foley catheter, is important within the junior medical team. This aims to secure the tract until an appropriate tube is available.

Similar cases with multiple advanced comorbidities had been reported previously with conservative management with eventual tube passage from the rectum [14,15] or had to undergo surgical correction with associated risks [16]. However, the presentations of similar scenarios and the aim for timely management remain challenging during the current COVID-19 pandemic.

The UK guidelines are clear regarding 14 days of isolation for severely immunocompromised patients in the context of COVID-19. The guidelines for mildly immunocompromised patients with multiple comorbidities are not clear, and so there is advice to review patients’ conditions [17]. The American College of Gastroenterology established and offered practical guidance for its members and even presented a webinar on this topic on April 27, 2020. They admitted some of these recommendations have been conflicting due to factors associated with the fast-changing situation; a lack of valid data; geographical differences in terms of prevalence; the availability of personal protective equipment; laboratory testing; and other resources [18]. Retrospective cohort studies by Zafar et al. have recently published work comparing the outcomes of COVID-19 mortality associated with high HbA1c levels, low Vitamin D levels, and low lymphocyte counts. They have concluded that there are more associations with increased mortality and the cumulative sum of comorbidities rather than absolute blood or serum counts for vitamin D levels and lymphocyte counts [19,20]. These researches point towards more association of comorbidity towards increased mortality.

We have proceeded to write about the need for awareness and early recognition of displaced GJ or PEG tubes with jejunal extensions, and its challenges for junior medical teams in an emergency department setting. This is with an aim to avoid potential structural, nutritional and metabolic consequences, including the emotional trauma, a patient has to endure while being managed in the acute medical setting. In the COVID-19 pandemic, it may be more appropriate to consider earlier submission of requests for invasive procedures, such as PEG insertion or jejunal extensions, by the necessary teams, for patients who are relatively immunocompromised.

## Conclusions

Invasive alternative feeding tubes such as GJ and PEG tubes with a jejunal extension (PEG-J) commonly get displaced or dislodged. Prompt recognition, investigation and timely management of associated clinical complications would ensure earlier patient discharge. During the COVID-19 pandemic, this is of particular importance for patients with multiple comorbidities, and who may be immunocompromised. 

In particular, the patients who are mild and moderate immunocompromised would benefit from clear guidelines of the National Institute for Health and Care Excellence (NICE) in the UK and The Institute for Clinical and Economic Review (ICER) in the USA. We hope, this case report would encourage more case reports and research work leading to the availability of management guidelines geared towards prompt procedures with timely discharge from the hospital, hence preventing the potentially immune-compromised patients from the risks of COVID-19 infection during COVID-19 pandemic times.

## References

[1] Freeman C, Ricevuto A, DeLegge MH (2010). Enteral nutrition in patients with dementia and stroke. Curr Opin Gastroenterol.

[2] Zuercher JN, Cumella EJ, Woods BK, Eberly M, Carr JK (2003). Efficacy of voluntary nasogastric tube feeding in female inpatients with anorexia nervosa. JPEN J Parenter Enteral Nutr.

[3] Gomes CA Jr, Lustosa SA, Matos D, Andriolo RB, Waisberg DR, Waisberg J (2010). Percutaneous endoscopic gastrostomy versus nasogastric tube feeding for adults with swallowing disturbances. Cochrane Database Syst Rev.

[4] Blumenstein I, Shastri YM, Stein J (2014). Gastroenteric tube feeding: techniques, problems and solutions. World J Gastroenterol.

[5] Kurien M, Leeds JS, Delegge MH (2013). Mortality among patients who receive or defer gastrostomies. Clin Gastroenterol Hepatol.

[6] Larson DE, Burton DD, Schroeder KW, DiMagno EP (1987). Percutaneous endoscopic gastrostomy. Indications, success, complications, and mortality in 314 consecutive patients. Gastroenterology.

[7] Itkin M, DeLegge MH, Fang JC (2011). Multidisciplinary practical guidelines for gastrointestinal access for enteral nutrition and decompression from the Society of Interventional Radiology and American Gastroenterological Association (AGA) Institute, with endorsement by Canadian Interventional Radiological Association (CIRA) and Cardiovascular and Interventional Radiological Society of Europe (CIRSE). Gastroenterology.

[8] Guloglu R, Taviloglu K, Alimoglu O (2003). Colon injury following percutaneous endoscopic gastrostomy tube insertion. J Laparoendosc Adv Surg Tech A.

[9] Friedmann R, Feldman H, Sonnenblick M (2007). Misplacement of percutaneously inserted gastrostomy tube into the colon: report of 6 cases and review of the literature. JPEN J Parenter Enteral Nutr.

[10] Bumpers HL, Collure DW, Best IM (2003). Unusual complications of long-term percutaneous gastrostomy tubes. J Gastrointest Surg.

[11] Hussien M, Fawzy M, Carey D (2001). Percutaneous endoscopic gastroscopy tube migration: a rare cause of a common surgical problem. Int J Clin Pract.

[12] Terlevich A, Hearing SD, Woltersdorf WW (2003). Refeeding syndrome: effective and safe treatment with phosphates polyfusor. Aliment Pharmacol Ther.

[13] Marinella MA (2003). The refeeding syndrome and hypophosphatemia. Nutr Rev.

[14] Polychronidis A, Karayiannakis AJ, Perente S, Botaitis S, Simopoulos C (2003). Enteral migration of a Pezzer tube after a feeding jejunostomy: report of a case. Surgery Today.

[15] Ozben V, Karataş A, Atasoy D, Sımşek A, Sarigül R, Tortum OB (2011). A rare complication of jejunostomy tube: enteral migration. Turk J Gastroenterol.

[16] Gupta V, Sharma AK, Pattnaik B, Kudria KC (2015). Spontaneous ante-grade enteral migration of jejunostomy tube: a rare complication. J Cancer Res Ther.

[17] (2022). Stepdown of infection control precautions and discharging COVID-19 patients and asymptomatic SARS-CoV-2 infected patients. https://www.gov.uk/government/publications/covid-19-guidance-for-stepdown-of-infection-control-precautions-within-hospitals-and-discharging-covid-19-patients-from-hospital-to-home-settings/guidance-for-stepdown-of-infection-control-precautions-and-discharging-covid-19-patients.

[18] (2022). The ACG Endoscopy Resumption Task Force: Guidance on safely reopening your Endoscopy Center. https://webfiles.gi.org/docs/policy/2020resuming-endoscopy-fin-05122020.pdf.

[19] Zafar M, Shahbaz M, Karkhanis M (2021). A retrospective observational study: is absolute lymphocyte count a prognostic marker in COVID-19?. Cureus.

[20] Zafar M, Karkhanis M, Shahbaz M (2022). Vitamin D levels and mortality with SARS-COV-2 infection: a retrospective two-centre cohort study. Postgrad Med J.

